# A compendium of community engagement responses to the COVID-19 pandemic

**DOI:** 10.1017/cts.2021.800

**Published:** 2021-06-14

**Authors:** Milton Mickey Eder, The PACER Group, Penny Ralston, Eugenia Millender, Joedrecka S. Brown Speights, Jessica De Leon, Sarah Wiehe, Gina Claxton, Dennis Savaiano, Cheryl Dennison Himmelfarb, Syed Ahmed, David Nelson, Jen Brown, Namratha Kandula, Darius Tandon, Ariel Thomas, Mona AuYoung, Wei-ting Chen, M. Kate Stewart, Robynn Zender, Arleen F. Brown, Savanna L. Carson, D’Ann Morris, Stefanie D. Vassar, Rodney Von Jaeger, Howard Taras, Francisco Tung Nguyen, Nynikka Palmer, Paula Fleisher, Abby Cabrera, Erica Wong, James Harrison, Mike Potter, Kevin Grumbach, Linda Cottler, Tamara Millay, Catherine Striley, Gia Mudd, Milton Eder, Karen Monsen, Robin Austin, Clarence Jones, Laura Sugarwala, John Cullen, Elissa Orlando, Nancy Bennett, Katrina Kubicek, Michelle Kipke, Sharon A. Croisant, Chantele Singleton, John Prochaska, Krista Bohn, Tamara A. Millay, Linda B. Cottler

**Affiliations:** 1 Department of Family Medicine and Community Health, University of Minnesota, Medical School, Minneapolis, MN, USA; 2 The PACER Group; Florida State University; Indiana University; Johns Hopkins University; Medical College of Wisconsin; Northwestern University; Scripps Health; Stanford University; University of Arkansas for Medical Sciences; University of California-Irvine; University of California-Los Angeles; University of California-San Diego; University of California-San Francisco; University of Florida; University of Kentucky; University of Minnesota; University of Rochester Medical Center; University of Southern California; University of Texas-Medical Branch; 3 Department of Epidemiology, Colleges of Medicine and Public Health and Health Professions, University of Florida, Gainesville, FL, USA

**Keywords:** Community engagement, community-engaged research, Translational Science, disparities, trust, COVID-19, CTSA, PACER

## Abstract

**Introduction:**

Clinical and Translational Science Award Program (CTSA)-funded institutions were charged with developing clinical and translational science programs and transforming clinical research at their institutions. Community engagement (CE) was recognized as a key component and catalyst of that transformation. CE hub capacities for working with communities and translating knowledge into practice have been illustrated through their COVID-19 responses.

**Methods:**

CE hub leaders met and discussed their CTSA’s early responses regarding the COVID-19 pandemic. The 2-hour discussion was distilled into themes which were sent to the CE hub leaders with a request for written accounts describing actions taken to engage local partners, communities, and institutions. The written reports form the basis for this compendium.

**Results:**

Eighteen institutions submitted written reports describing activities in relation to six themes: (1) listen to the community and respond to concerns, (2) collect data to understand the impact of COVID-19 on distinct communities and groups, (3) communicate science and address misinformation, (4) collaborate with health departments, (5) engage hubs and underrepresented populations in COVID-19 research, and (6) support our own well-being and that of others.

**Conclusions:**

Bidirectional interactions comprise the foundation of CE, which requires trusted partnerships that sustain communication through a series of activities and goals. The nimble responses to the pandemic substantiate the need for CE programs to maintain the infrastructure necessary to achieve the primary CTSA goals of improving health within and across communities and localities as well as expanding research participation of community members.

## Introduction

The National Institutes of Health (NIH) Clinical and Translational Science Award Program (CTSA) was launched in 2006 with a primary mission to “more efficiently translate the rapidly evolving knowledge developed in basic biomedical research into treatments to improve human health.” The CTSA project included transforming academic institutions by studying clinical and translational science and developing its potential as an academic discipline. As a key component of clinical and translational science, the community engagement (CE) core aims were limited “to enhancing public trust in clinical and translational research, and facilitating recruitment of research participants from the community.”

NIH initiatives preceding the CTSA program were also expected to build trust and increase clinical research recruitment from diverse communities (e.g., National Institute of Environmental Health Sciences (NIEHS)/Environmental Protection Agency Children’s Environmental Health and Disease Prevention Research Centers, the NIEHS Superfund Research Program, NIH Director’s Council of Public Representatives). The Cancer Clinical Oncology and the Minority-based Cancer Clinical Oncology were programmatic attempts to diversify enrollment. By comparison to CTSAs, these programs did not as clearly involve increasing access to new therapies, increasing primary care involvement, or improving population health outcomes.^1,2^

The CTSA awards created new expectations and new opportunities for institutions to meaningfully engage racially, ethnically, and culturally diverse, rural and urban populations. CTSA institutions were asked to build on their strengths and develop team-based approaches to maximize the value of clinical research for populations with a high prevalence of comorbid diseases such as asthma, cardiovascular disease, depression, diabetes, and hypertension.

Over 15 years of CTSA funding, tremendous progress can be observed in reaching beyond institutional walls and sociological boundaries to engage communities and build trust in local translational research enterprises. Many of the 60 CTSA institutions are situated at ground zero for systemic inequalities where a single mile predicts more than a 10-year difference in life expectancy.^3^ CTSAs have worked to expand access to clinical trials while also demonstrating the value of community relationships to enhance the institutional capacity both to conduct clinical trials and to use knowledge for human benefit. CTSAs have engaged communities to participate in addressing water crises, food insecurities, and natural disasters (fires, hurricanes, tornadoes, etc.).

CTSA CE programs have been expected to build trusting relationships with community members and partners. While trust has neither been consistently nor uniformly assessed across institutions, it has been thought to have been built and sustained. In addition, even the word trust has morphed into trustworthiness over time, suggesting that CE programs are sustaining strategic dialogues with communities. Responsibility for health outcomes has too often been considered the lone responsibility of CE programs, when it should also be recognized as the logical outcome of a successful translational science program and consequently the responsibility of the CTSA hub.

COVID-19, the disease caused by the virus SARS-CoV-2, has disproportionately infected populations across the USA that has historically endured myriad health disparities and has disrupted clinical research, further reduced access to health care in underresourced communities, and challenged trusting partnerships. Increased susceptibility to COVID-19, along with health literacy deficiencies and inaccurate messaging about the available scientific evidence, exacerbated hospitalizations and deaths within the communities and among the people CE programs serve.

Many CTSA hubs have established partnerships within communities, which enhanced their potential to provide a timely response to health crises such as COVID-19. Paralleling the initial synergy paper, discussing how the already built CTSA infrastructure could be used to address the nation’s opioid crisis,^4^ this paper recounts how CTSA CE cores worked collaboratively with community partners to implement clinical research and public health initiatives that responded to the health challenges presented by COVID-19.

This compendium is based on accounts provided by members of Partners for the Advancement of Community Engaged Research (PACER), a Special Interest Group of the Association of Clinical and Translational Science (ACTS). For the past 6 years, PACER has organized monthly meetings, bringing individuals focused on CE together. PACER has 201 members who are affiliated with 80 institutions and community organizations in 34 states; membership is independent of CTSA status. PACER has no limit on the number of participants from each hub and no restrictions on what can be discussed at meetings, unlike CTSA “enterprise” groups. A typical meeting agenda begins with new participant self-introductions, followed by a presentation and group discussion, with time to introduce and assess interest in new research ideas. Meetings close with miscellaneous announcements and acknowledgements (information about local programming, particularly those that support virtual participation like the University of Florida’s (UFL) *Our Community Our Health*). PACER consistently encourages collaborative learning and project planning among its members. This compendium further illustrates how PACER members at 18 institutions collaborated with their local communities during the pandemic to facilitate public health initiatives.

## Methods

PACER holds an annual meeting to review its mission and plan future activities in coordination with the national ACTS meeting. The 2020 meeting in early April was by necessity virtual as part of the effort to act responsibly and “flatten the pandemic curve.” The meeting began with an explanation that a paper was being considered on the agenda’s main topics – maintaining and continuing to build trust with and for our communities; identifying current community needs and services for vulnerable populations; addressing loneliness, isolation, and other mental health issues; keeping research groups together during a research hiatus when programs and services were suspended. A summary of the initial synergy study was put forth as a potential model for the proposed paper.^4^ During the 2-hour meeting, attendees described interactions with local community partners and how they were working with communities to identify and address immediate needs. The virtual meeting included about 30 investigators/community partners from 20 CTSA hubs; 18 institutions subsequently contributed written materials to support this publication (Table 1).

**Table 1.** PACER group members contributing written reports

Institution	Individual Contributors
Florida State University	Penny Ralston, Eugenia Millender, Joedrecka S. Brown Speights, and Jessica De Leon
Indiana University, Purdue University, University of Notre Dame	Sarah Wiehe, Gina Claxton, and Dennis Savaiano
Johns Hopkins University	Cheryl Dennison Himmelfarb
Medical College of Wisconsin	Syed Ahmed and David Nelson
Northwestern University	Jen Brown, Namratha Kandula, Darius Tandon, and Ariel Thomas
Scripps Health	Mona AuYoung
Stanford Medicine, Office of Community Engagement	Wei-ting Chen
University of Arkansas for Medical Sciences	M. Kate Stewart
University of California – Irvine	Robynn Zender
University of California – Los Angeles	Arleen F. Brown, Savanna L. Carson, D’Ann Morris, and Stefanie D. Vassar
University of California – San Diego	Rodney Von Jaeger and Howard Taras
University of California – San Francisco	Nynikka Palmer, Paula Fleisher, Abby Cabrera, Erica Wong, James Harrison, Mike Potter, Kevin Grumbach, and Tung Nguyen
University of Florida	Linda Cottler, Tamara Millay, and Catherine Striley
University of Kentucky	Gia Mudd
University of Minnesota	Milton Eder, Karen Monsen, Robin Austin, and Clarence Jones (Hue-MAN Partnership)
University of Rochester Medical Center	Laura Sugarwala, John Cullen, Elissa Orlando, and Nancy Bennett
University of Southern California	Katrina Kubicek and Michelle Kipke
University of Texas Medical Branch	Sharon A. Croisant, Chantele Singleton, John Prochaska, and Krista Bohn

PACER, Partners for the Advancement of Community Engaged Research.

The two PACER cochairs (LC and ME) and the PACER administrative staff member (TAM) reviewed the recorded meeting transcript and distilled the meeting transcript and their personal notes into seven themes. The themes were incorporated into a summary statement circulated to all PACER members with an open request for descriptions of hub activities that related to identified or additional themes. The request further explained that any patterns identified in how CTSA hubs mapped activities in relation to goals would be portrayed as illustrating common approaches to CE. Members were encouraged to explain the metrics they would use to assess impact of their strategies. Finally, a request was made for supplementary materials for further dissemination to increase collaborations.

Materials submitted were analyzed using a semiotic approach, which focuses on the relationship between the language (i.e., sign) describing activities and the meaning (i.e., signifier) derived from the actual language used. A semiotic approach would suggest that the signs used to create a message may be discordant with the message’s perceived meaning. This study relied on an iterative process in which the relationship between sign and signifier^5,6^ was continuously examined to achieve unanimous approval of the themes shared following the PACER meeting. Accounts of activities were discussed by email to confirm the thematic relationship to the activities described. In addition, manuscript versions were circulated to all the authors to further validate the organization of activities by theme.

## Results

Reports from institutions specified a connection between the themes of listening and responding to the community; this reduced the seven themes to six. In the sections below, we summarize key findings related to each theme. The examples provided in the tables only occasionally describe metrics and evaluation plans.

### Listen to the Community and Respond to Concerns

The hub reports universally joined what were initially two distinct themes. Combining listening and responding to the community situates bidirectional communication at the foundation of engagement. Ongoing communicative interaction enabled increased awareness of and access to community perceptions and issues and informed all the themes reported in this paper (Table 2).

**Table 2.** Examples of institutional approaches to listening and responding to the community

Institution	Theme One Activities
Florida State University	Convened a 24-member stakeholder group from Gadsden and Leon Counties and hosted a community led town hall for sharing personal stories about COVID-19 experiences
Indiana University Clinical and Translational Science Institute	Held state-wide virtual meetings with community and university partners
Northwestern University	Funded community-based organizations to continue addressing community needs and facilitated virtual discussions about community engagement and equity in research during the pandemic^7^
SOCCER Consortium	Conducted town halls, individual interviews, and listening sessions with community partners
Stanford Medicine Office of Community Engagement	Joined six counties in a coalition concerned with economic and social displacement of residents
University of California – Irvine	Hosted Community Engagement Studios
University of California – Los Angeles	Convened best practices for community engagement with community partners, resulting in a partnered manuscript^8^
University of Florida	HealthStreet worked in the parking lot to distribute meals, clothing, toiletries, and feminine hygiene products – things the community said they needed right now
University of Kentucky Center for Clinical and Translational Science Community Engagement and Research Core	Worked with Latinx community leaders to initiate a multi-media educational campaign; involved community health workers and responded to needs in collaboration with Community Advisory Boards and community-based partnerships
University of Texas Medical Branch	Research, Education, And Community Health Coalition (REACH) facilitated community access to academic resources engaged around COVID-19-related issues^9^

SOCCER, Southern California Consortium of Community Engagement Resources (University of California – Irvine, University of California – Los Angeles, University of California – San Diego, and University of Southern California, Scripps Health).

Hub reports described involvement by Community Advisory Boards and Councils, participation by other hub centers and institutes, and contributions in the form of connections to established community partners. The members also described participating in coalitions involving community organizations and state, county, and city health departments as well as other agencies. Hub approaches to engagement with COVID-19 coalesced around two strategies: those led and managed by CTSA hubs and those in which hub representatives were among the annual meeting attendees.

### Collect Data to Understand the Impact of COVID-19 on Distinct Communities and Groups

Data collection and analyses are integral to research, and this activity was by necessity an anchor for engagement through the CE cores of the clinical and translational hubs. Data gaps regarding COVID-19 cases were noted early in the pandemic, particularly a lack of data concerning what turned out to be significant disparities in community disease burden.^10–12^ Data collected by hubs were generated by conducting interviews with partners and hosting CE Studios to learn directly from community members (Table 3). A few hubs added questions to regularly administered surveys or developed new surveys that community partners then circulated. At the UFL, the CE program (HealthStreet) contacted their 12,000 members to keep in touch through a series of questions on COVID-19 testing, vaccine hesitancy, food insecurity, loneliness, stress, and health needs; to date, over 3000 members have been interviewed. The data have been presented citywide to generate more testing sites, improve access to care, and provide other needed services.

**Table 3.** Examples of collecting data to understand how COVID-19 impacted local communities and groups

Institution	Theme Two Activities
Indiana University Clinical and Translational Science Institute	Facilitated a COVID-19 registry to track health, housing, and economic impacts; amended a community asset survey to gather data on COVID-19 and associated health-care delivery
Scripps Health and the University of California – Los Angeles	Added questions to a phone survey to learn about concerns with visiting medical offices
Stanford Medicine Office of Community Engagement	Developed a COVID-19 Community Outcomes Survey (COCO) in English, Spanish, Vietnamese, and Chinese to identify and share information about unmet needs in distinct minority populations^13^
University of Arkansas for Medical Sciences	Assessed COVID-19 homeless shelter needs and practices and worked with the health department to address their needs
University of Florida	Worked with City Commissioners to get COVID case data by zip code for race, sex, and age; HealthStreet instituted a Reconnect Phone Survey to understand the impact of COVID-19 and vaccine hesitancy on their members
University of California – Los Angeles	Conducted COVID-19 vaccine hesitancy and acceptability focus groups with communities facing a high burden of COVID-19 (race/ethnicity, age, and low income)
University of Kentucky	Appalachian Translational Research Network partners developed a survey to assess the impact of COVID-19 across Appalachia and acceptance of a vaccination
University of Minnesota	Added COVID-specific questions to a survey about social determinants of health

Data collection activities and goals were reported to be a recurring meeting agenda item at numerous hubs. Data availability and review led hub advisory boards and community coalitions to increase their meeting frequency to address quickly changing conditions. The emphasis on data-informed decision-making represented a combination of translational research and quality improvement activities. Data provided insights into local community conditions and were considered essential for evaluating the programmatic responses developed to address emergent local health conditions. While recognizing the importance of applied research as necessary to fulfilling the goal of translating knowledge into improved care delivery and outcomes, these reports on early initiatives to address pandemic-generated public health issues did not necessarily include plans to address or change local policy. In addition, the efforts to amass data and confront the pandemic in real time were described separately from hub efforts to build COVID-19 research recruitment registries in support of their clinical and translational research responsibilities.

### Communicate Science and Address Misinformation

Community partners and community members indicated dissatisfaction with the changing, confusing, and sometimes contradictory COVID-19 information available; they also shared their enthusiasm and need for access to science-based information about the nature of the virus and ways to stay healthy. CE scientists were asked to post to blogs, contribute to weekly newsletters, appear at virtual events, and join providers at COVID-19 testing sites to foster trust in the testing process. Hubs organized COVID-19 community virtual town halls to provide access to researchers and to inform local government and community officials on the available scientific evidence (Table 4). To not only “talk the talk but walk the walk,” CE program staff organized medical students, City Commissioners, Mayors and others, to share credible information, to contribute to the development of dashboards, to explain the importance of incidence data by zip code and race, and to disseminate personal protective equipment (PPE).

**Table 4.** Examples of communicating science and addressing misinformation

Institution	Theme Three Activities
Indiana University Clinical and Translational Science Institute	Hosted town halls and created infographics and media notices about Personal Protective Equipment via social media
Johns Hopkins University	Coordinated and shared information produced across the institution with community partners
Medical College of Wisconsin	Relied on prior disaster preparedness research and community partners, school districts and government offices to reach underrepresented populations
Northwestern University	Developed databases to facilitate idea and resource sharing to address community needs
Stanford Medicine Office of Community Engagement	>1600 registrations for a live event to share information related to COVID-19^14^
University of Arkansas for Medical Sciences	Fay W. Boozman College of Public Health (COPH) stood up a COVID-19 risk communication working group, including Arkansas Faith Academic Initiatives for Transforming Health (FAITH) Network.
University of California – San Diego	Issued a web-based guide for community members to evaluate information in news stories and conducted town halls on COVID-19
University of California – Los Angeles	Published multiple COVID-19-related articles in local ethnic media, including La Opinion (in Spanish) and the Los Angeles Sentinel (African American readership)^15^
University of California – San Francisco Clinical and Translational Science Institute	Collaborated with the University of California–San Francisco Latinx Center of Excellence and Latinx community coalitions, hosted webinars in Spanish and English to address urgent community information needs
University of Florida	Worked with the City Commissioner and Community Advisory Board Chair to present early data from the COVID-19 survey in community contexts, including an Our Community Our Health event titled *Pandemic on Top of Epidemics*
University of Rochester Medical Center	Organized virtual COVID-19 team science sessions with community participants and discussions on the Rochester and Finger Lakes Public News Station; developed a “Six Feet Saves” campaign with Spanish and English messaging and a local dissemination plan^16^
University of California – Los Angeles and University of Southern California	Held community conferences about COVID-19 and related resources for community health workers and *Promotoras*
University of Texas Medical Branch	Hosted town halls and developed infographics, media notices about personal safety; used social media to hold conversations on difficult issues (e.g., cruelty of policies that isolate families from their loved ones at time of death)

Hub CE partnerships also engaged in sessions to discuss the confusion and suspicion regarding the role of race in health disparities and frustration with the scientific process, particularly regarding current therapies and vaccine development. These conversations were reported to have altered team member perceptions of CE’s role in balancing research, interpersonal, and partnership dynamics.

### Collaborate with Public Health Departments

Several institutions strengthened existing relationships with local health departments, primarily by participating in coalitions (Table 5). Hubs organized registries to collect data at multiple time points for surveillance purposes in tandem with or for health departments. The awareness of data gaps and racial disparities in outcomes led those developing the registries to include health, economic, behavioral, and exposure information. Some hubs set up their registry to support recruitment for COVID-19 and other health disparities research. Hub coalition partners reported expanding their reach into secondary partner networks to increase registry enrollment. Some registries included multiple interfaces to accommodate participation across language differences (e.g., Spanish, Vietnamese, and simplified Chinese). In addition, some hubs became the center of activity for contact tracing. At many hubs, the CE infrastructure was instrumental in helping with or in alleviating the burden on Departments of Health.

**Table 5.** Examples of collaborating with public health departments

Institution	Theme Four Activities
University of California – Los Angeles Clinical and Translational Science Institute	Collaborated with the Department of Human Services on two Patient-Centered Outcomes Research Institute (PCORI) grant submissions
University of California – San Francisco	Collaborated with San Francisco Department of Health and community coalitions to set up community-based testing events in neighborhoods with high hospitalization rates, collecting and sharing population health data
University of Kentucky	Coordinated initiatives with their Department of Health
University of Minnesota	Community collaborated with the School of Nursing to collect data on community COVID-19 knowledge and attitudes to inform Department of Health programming
University of Rochester Medical Center	Center for Community Health and Prevention staff partnered with the Monroe County Department of Public Health to collect, analyze, and share data locally and nationally^17^
University of Texas Medical Branch	University of Texas Medical Branch at Baylor-Rice and the Gulf Coast Center for Precision Environmental Health promoted the Greater Houston COVID-19 Registry^18^

### Engage Hubs and Underrepresented Minorities in COVID-19 Research

Institutions across the consortium have consistently succeeded at engaging community partners in research; in some hubs, this has been accomplished through pilot study funding. One common response to the pandemic reported by hubs was an almost immediate designation of pilot funds for community engaged COVID-19 research. The seriousness of COVID-19 made it particularly important to work with established partners to immediately engage communities in the research enterprise. While ongoing human subjects research was suspended for some time due to additional risks of physical interaction posed by the infectious COVID-19, projects relevant to mitigation and treatment of COVID-19 were prioritized (Table 6). To advance project planning, investigators incorporated social (or physical) distancing using Zoom and conference calls for community meetings. These options were not always available or desirable; UFL reported being able to continue meeting with community members and partners in their parking lot, so that they could meet face-to-face while adhering to CDC social distance guidelines.

**Table 6.** Examples of hubs engaging underrepresented minorities in COVID-19 research

Institution	Theme Five Activities
Johns Hopkins University	Community Research Advisory Council provided input on study design, implementation and communications, developed Hope Registry to support COVID-19 study
SOCCER	Multi-institutional partnership with local COVID-19 research response groups engaged community expertise in devising and conducting COVID-related research, including COVID-19 vaccine clinical trials
Stanford Medicine Office of Community Engagement	Partnered with community for research recruitment in diverse racial/ethnic, especially Latinx communities
University of Arkansas for Medical Sciences	Provided technical support in developing COVID-19-related community-engaged research
University of Florida	Continued to recruit participants to its HealthStreet cohort for linkage to all University of Florida research studies
University of California-San Francisco	Assembled a dedicated COVID-19 Research Patient and Community Advisory Board
USC	Worked with its Filipino community advisory board to transform an in-person randomized control trial to online participation, which was projected to allow a broader reach in California

SOCCER, Southern California Consortium of Community Engagement Resources (University of California-Irvine, University of California – Los Angeles, University of California – San Diego, University of Southern California, Scripps Health)

Enduring CE partnerships based upon trust and maintained by ongoing bidirectional communications enabled institutions to launch numerous initiatives. However, University of California – San Francisco’s (UCSF) COVID Research Patient and Community Advisory Board (COVID Research PCAB)^19^ uniquely advanced clinical and translational research by integrating stakeholders from across institutional research initiatives. The UCSF COVID Research PCAB was quickly established as a supplement to the existing UCSF Accelerating Systematic Stakeholder, Patient and Institution Research Engagement Stakeholder Advisory Board, a Patient Centered Outcomess Research Institute Eugene Washington Engagement Awardee. Community members from the CTSI Integrating Special Populations and project-specific community advisory boards were also recruited. UCSF assembled patient and community advisors from populations underrepresented in research. Experienced members of the COVID Research PCAB recommended strategies for patient and community stakeholder engagement and advocated for health equity. UCSF uniquely coordinated experienced community and stakeholder input at a pan-institutional level to contribute to the review of all proposed COVID-related research.

Other hubs engaged members from populations underrepresented in advising on COVID-19 research activities (e.g., recruitment, data collection, retention, implementation, and dissemination strategies). The coordination of community input and establishment of the COVID Research PCAB exemplified a best-practice institutional transformation expected for a CTSA funded institution.

### *Support Our Own Well*-*being and That of Others*

The final theme explored the need for CE professionals to support their own personal health and well-being. For example, Indiana University and the UFL hosted weekly zoom social hours (non-alcoholic) with staff, faculty, and students together to foster interaction among their teams and reduce stress. The Southern California Consortium of Community Engagement Resources hubs reported outreach to community partners and individuals, actively encouraging sensitivity regarding mental health to support resilience among researchers, research teams, partners, research and community participants. Finally, the University of Rochester Medical Center contributed to an NIH/NCATS initiative to collect and disseminate mental health and wellness resources for trainees and scholars in response to COVID-19.

## Discussion

The reports on institutional activities in relation to each of the CE themes illustrate both variability in local strategies for engaging community partners and the imperative of developing a hub infrastructure to sustain ongoing dialogues. The descriptions of community-academic bidirectional engagement during the early weeks and months of the COVID-19 pandemic collectively demonstrate the importance of listening to community issues and organizing a response that can quickly coordinate research and community resources and expertise. Institutions that possessed an infrastructure capable of collecting, organizing, and sharing information did so by adding questions to existing community surveys or launching new efforts to gather and share information with communities about needs due to COVID-19. Reports from CTSAs indicated a strong ongoing commitment to interdisciplinary team science and a willingness to expand community member and other stakeholder involvement in institutional partnerships even at this time.^20–22^ The reports informing this manuscript indicate that academic institutions have augmented their infrastructure for responding to community issues instead of focusing on individual, isolated research projects.^23–27^

Especially now, further transformation is necessary for institutions to develop the capacity to engage communities in all phases of translational science. This aligns with the NCATS Advisory Council Working Group calling for a workforce that is competent in CE and collaboration.^28^

Honoring relationships with and responsibilities to community partners was among the issues expressed throughout the PACER virtual call. While trust was often mentioned, the discussion did not consider if community trust in researchers and their individual projects could be assessed in the same way as trust in the institution itself.^29,30^ The CE activities responding to the pandemic provide new opportunities for understanding our trustworthiness in the eyes of community members. As institutions continue to transform and expand CE within their clinical and translational research programs, incorporating trust types into the evaluation could greatly advance the science of CE.

## Limitations

This compendium is based on the voluntary submission of reports with an analysis that emphasized the similarities in descriptions of CE activities during a pandemic. The primary goal was to identify the range of hub activities. Hub reports did not describe disruptions to ongoing research activities, which may reflect the study focus on responses to the pandemic.

This paper includes the PACER Group as one of its authors to recognize the members who contributed not only to the development and implementation of activities in response to the pandemic but who contributed to the development of this paper (Table 1). PACER members represent a national network of CE scientists, many of whom meet monthly to share ideas and valued approaches. While ideas regarding authorship will only be further complicated as community members participate in team science, hub reports did not consistently describe their communication processes, obscuring the value of local dissemination as contributing to the goals of diversifying recruitment and improving health.

## Conclusion/Next Steps

This compendium organized hub reports in the pursuit of one shared goal: to not turn our backs on the communities we have been working with – communities with which we have established trusting relationships. The pandemic provided new stories about CE. It has reminded us that we are all members of communities and that more than ever we must continue to engage other communities and sustain partnerships. Collectively, the reports suggest that the translational science business model must support both community-engaged research projects and the engagement of community voices and perspectives in facilitating institutional transformation to become translational science centers.

The range of institutional responses to local pandemic challenges suggests hubs vary in establishing and supporting partnerships and in their capacity to engage communities. While sharing successes, these hub reports also illuminate the importance of translational science developing the capacity to support communication networks and the programs to generate content. As evaluation of CE assesses diversity in research recruitment and participation, it has the additional opportunity to assess the role of communication with and for communities in improving health and promoting equity as measured by reductions in disparities.
